# Getting into hot water: sick guppies frequent warmer thermal conditions

**DOI:** 10.1007/s00442-016-3598-1

**Published:** 2016-03-10

**Authors:** Ryan S. Mohammed, Michael Reynolds, Joanna James, Chris Williams, Azad Mohammed, Adesh Ramsubhag, Cock van Oosterhout, Jo Cable

**Affiliations:** School of Biosciences, Cardiff University, Cardiff, CF10 3TL UK; Department of Life Sciences, Faculty of Science and Technology, University of the West Indies, Mona, Trinidad and Tobago; National Fisheries Services, Environment Agency, Brampton, Cambridgeshire PE28 4NE UK; School of Environmental Sciences, University of East Anglia, Norwich Research Park, Norwich, NR4 7TJ UK

**Keywords:** Behavioural fever, Climate change, Thermal gradients, Trinidadian guppy, *Gyrodactylus*

## Abstract

Ectotherms depend on the environmental temperature for thermoregulation and exploit thermal regimes that optimise physiological functioning. They may also frequent warmer conditions to up-regulate their immune response against parasite infection and/or impede parasite development. This adaptive response, known as ‘behavioural fever’, has been documented in various taxa including insects, reptiles and fish, but only in response to endoparasite infections. Here, a choice chamber experiment was used to investigate the thermal preferences of a tropical freshwater fish, the Trinidadian guppy (*Poecilia reticulata*), when infected with a common helminth ectoparasite *Gyrodactylus turnbulli,* in female-only and mixed-sex shoals. The temperature tolerance of *G. turnbulli* was also investigated by monitoring parasite population trajectories on guppies maintained at a continuous 18, 24 or 32 °C. Regardless of shoal composition, infected fish frequented the 32 °C choice chamber more often than when uninfected, significantly increasing their mean temperature preference. Parasites maintained continuously at 32 °C decreased to extinction within 3 days, whereas mean parasite abundance increased on hosts incubated at 18 and 24 °C. We show for the first time that gyrodactylid-infected fish have a preference for warmer waters and speculate that sick fish exploit the upper thermal tolerances of their parasites to self medicate.

## Introduction

Temperature is perhaps the most important environmental determinant of the activity and performance of ectothermic vertebrates, and is particularly critical for fishes that, unlike amphibians and reptiles, are inefficient thermoregulators (Atkinson 1994). Fish behaviourally regulate their body temperature by selecting habitats with thermal regimes that optimise physiological performance (Reynolds et al. 1976; Ward et al. 2010). The metabolism, feeding rate and activity levels of ectotherms generally increase with temperature until conditions become stressful. Thermal stress can have long-lasting effects on fish behaviour with respect to migration (Jonsson and Jonsson 2009), reproductive success (Pankhurst and Munday 2011), predatory avoidance (Marine and Cech 2004), and shoaling (Weetman et al. 1998, 1999). For temperate fish, this results in marked seasonal and diel behaviours, but even tropical species are subjected to distinct temperature heterogeneities (Webb et al. 2008).

In addition to optimizing physiological performance, ectotherms exploit thermal regimes to hinder parasite transmission and development. A change in a host’s thermal preference driven by pathogenic infection, otherwise known as ‘behavioural fever’, has been documented in several taxa including bumblebees (Müller and Schmid-Hempel 1993), locusts (Elliot et al. 2002), lizards (Vaughn et al. 1974) and fish. The first evidence of behavioural fever in fish was observed in largemouth bass (*Micropterus salmoides*) and bluegill sunfish (*Lepomis macrochirus*); both species displayed a significant increase (+2.7 °C) in mean temperature preference when inoculated with bacteria (Reynolds et al. 1976). This response was associated with bacterial pyrogens (fever-inducing chemicals) acting directly on the host’s hypothalamic thermoregulatory centre (Reynolds et al. 1976). A subsequent study speculated that an increase in thermal preference by the fish host up-regulates the immune response against parasite infection (Covert and Reynolds 1977). Using zebrafish (*Daniorerio*) infected with viraemia of carp virus it was confirmed that host behavioural fever induces a major up-regulation of the innate immune response, in this case expression of anti-viral genes, which subsequently cleared viral infections within infected fish (Boltaña et al. 2013).

Acute thermal changes can be detrimental to the immune functions of fish (reviewed in Martin et al. 2010). However, some immune responses including elevations in lysozyme and immunoglobulin M levels are positively correlated with temperature until thermal limits are exceeded (Bowden et al. 2007; Marcos-Lopez et al. 2010). Thermal stress can reduce host immunocompetence thereby increasing disease susceptibility in ectotherms (Rohr and Raffel 2010). Interactions between these factors ultimately determine whether infections lead to severe pathology and even mortality, or host recovery. Parasites also respond directly to thermal variation, as elevated temperatures typically reduce development time. For example *Schistocephalus solidus* pleroceroid larvae, infecting three-spined sticklebacks, have faster growth rates and become infectious to their definitive host sooner at 20 °C compared to 15 °C (Macnab and Barber 2011).

For directly transmitted ectoparasites, including monogenean gyrodactylids, the rate of reproduction is positively correlated within a temperature range from 17 to 28 °C in tropical gyrodactylids, and 2.5–19.5 °C in temperate species (Scott and Nokes 1984; Jansen and Bakke 1991). Gyrodactylids are ubiquitous on teleosts, feeding on the skin and fin tissues of a host (Kearn 1996; Harris et al. 2004). Their life history traits, transmission and population dynamics have been extensively studied using the Trinidadian guppy-*Gyrodactylus* system (reviewed by Cable 2011). *Gyrodactylus turnbulli,* a common guppy ectoparasite, exhibits a viviparous reproductive strategy (Cable and Harris 2002), often resulting in explosive population growth, which can significantly impede host survival (e.g. Cable and van Oosterhout 2007a). As gyrodactylid embryonic development is temperature dependent (reviewed by Bakke et al. 2007), natural variations in water temperature can determine parasite population growth.

Whilst guppies exhibit broad temperature tolerance (Reeve et al. 2014), small changes in water temperature can dramatically modify gyrodactylid life history traits (Bakke et al. 2007), and temperatures exceeding 30 °C impede *G. turnbulli* survival (Scott and Nokes 1984). The present study investigates the thermal preferences of guppies in female-only and mixed-sex shoals, when uninfected and infected with the ectoparasite *G. turnbulli*. We also examine the temperature tolerance of these parasites by monitoring population trajectories on fish maintained at constant temperatures of 18, 24 or 32 °C. We hypothesize that guppies infected with *G. turnbulli* will frequent warmer water, in comparison to when they are uninfected, and exposure to extreme thermal conditions has benefits in terms of self-medication against parasites.

## Materials and methods

### Host and parasite origin

Guppies from the Lower Aripo River, Trinidad, were collected in 2010 and stock populations were housed in breeding tanks at 24 ± 0.5 °C at Exeter University. Fish were transferred to Cardiff University in April 2012 where they were maintained in 120-L aquaria at 24 ± 0.5 °C and fed daily on Aquarian® tropical fish flakes and occasionally frozen bloodworm.

The Gt3 strain of the parasite *G. turnbulli* was isolated from ornamental pet shop guppies in 1997, and was maintained on small numbers of fish (four to six individuals) in laboratory cultures at 24 °C under a 12-h light:12-h dark lighting regime. To prevent parasite extinction, each culture pot was subsidized with naïve fish twice weekly. Each pot contained a minimum of four culture fish collectively infected with approximately 40 gyrodactylid worms. To quantify mean parasite abundance (the total number of worms/the number of hosts including zero counts), guppies were anaesthetized with buffered 0.02 % tricaine methanesulfonate (MS222) and screened using a dissecting microscope with fibre optic illumination. For experimental infections, a heavily infected donor fish from the parasite culture was sacrificed and two to six worms transferred through direct contact onto the caudal fin of an anaesthetized recipient fish, as observed using a dissecting microscope. To remove parasites, fish were chemically treated using 0.1 % dilution of Levamisole (Norbrook, UK), and subsequently screened weekly over 3 consecutive weeks to ensure that the infection had been eliminated (see Schelkle et al. 2009).

### Experimental set-up

**Fig. 1:**
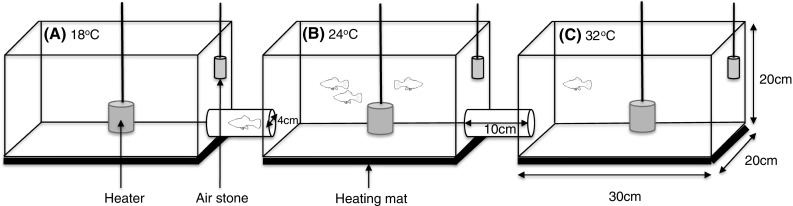
The experimental arena consisted of three aquaria interconnected by two plastic tubes (10 cm length × 4 cm diameter). The side walls of the arena were covered with black paper to reduce disturbance to the fish, with one side left open for observations. Tanks were filled with dechlorinated water to a depth of 15 cm. Placing air stones, aquarium heaters and heating mats in and under each chamber established a thermal gradient across the arena, and a consistent temperature within each tank. **a** Chamber A was maintained at 18 °C, **b** chamber B at 24 °C, and **c** chamber C at 32 °C, each ±0.5 °C. **b** Fish were always introduced and returned to chamber B during an experimental trial

The experimental arena consisted of three plastic aquaria (30 × 20 × 20 cm) connected by two plastic tunnels (10 cm length × 4 cm DIA; Fig. 1). All tanks were filled with dechlorinated water to a depth of 15 cm. The apparatus was covered by black paper on five sides to reduce disturbance by external stimuli, with one side left open to allow observations. The experiment was conducted in a temperature-controlled room (15 ± 0.5 °C), under a 14-h light:10-h dark lighting regime. Across the arena, a temperature gradient was established using heating mats under the central chamber (chamber B), and an aquarium heater in one side chamber (chamber C). Chambers A and B also contained small aquarium heaters that were not switched on to ensure uniform conditions within each chamber. Chamber A was maintained at 18 ± 0.5 °C, chamber B at 24 ± 1 °C and chamber C at 32 ± 0.5 °C. Each chamber was uniformly aerated to prevent a thermocline developing within the tank.

### Experimental procedure

Female-only (five females) or mixed-sex (three females, two males) shoals (*n* = 14 per shoal type) were placed in a 30 × 15 × 15-cm aquarium to familiarise for 7 days prior to an experimental trial (according to Richards et al. 2010). Females typically form small shoals in the wild, between which males move in search of mating opportunities (Griffiths and Magurran 1998), hence the rationale for using female-only and mixed-sex shoals with natural sex ratios in the current study. Using a crossover experimental design, shoals (six female only and six mixed sex) were infected at the start of the trial and monitored for 2 days. On day 3, these fish were artificially cleared of parasites and observed for a further 2 days. The remaining shoals (eight female only and eight mixed sex) started trials with uninfected individuals and were subsequently infected on day 3. Thus, each shoal served as its own infected/uninfected control, whilst the crossover design controlled for the potential effect of time by alternating the infection point. Experimental infections were conducted by housing each shoal with a heavily infected donor fish (>200 Gt3 worms) for 2 days in an infection tank (20 × 10 × 10 cm). Fish were then anaesthetized, and their gyrodactylid intensities recorded. To achieve a moderate infection (mean gyrodactylid intensity 22.5; range 13–34) per fish, additional worms were manually transferred to the caudal fin of some hosts from an infected donor fish (as previously described). Control fish were sham infected, whereby they endured the same handling and period of anaesthetisation, but were not exposed to parasites.

During a trial, either a female-only or mixed-sex shoal was introduced into chamber B of the arena. Profiles were created for each fish within a shoal documenting unique body colouration and markings enabling individuals to be distinguished. Over two consecutive days, each individual was observed five times per day with 2-h intervals in between. During a focal follow, fish in each chamber (A, B or C) were recorded every 10 s for 1 min, accumulating 10 min of observational data per fish. Individuals were then removed from the arena by scooping them up in a plastic 10 × 10-cm container to prevent parasite dislodgement, anaesthetised using 0.02 % MS222 and screened for *G. turnbulli* using a dissection microscope with fibre optic illumination. Fish were then either experimentally infected, or their parasites chemically removed, thereby reversing their infection status. Following chemical treatment, individuals were screened to ensure no parasites remained on the host. At the end of a trial, these fish were screened twice more to ensure they were not infected, to confirm that they were parasite-free during the final days of observation. Uninfected fish were also exposed to levamisole and anaesthetic to account for their potentially confounding effects on fish behaviour. Fish monitoring was then resumed for a further 2 consecutive days. For these trials, fish were again introduced into chamber B of the experimental arena and allowed to habituate for 2 h. This design allowed us to compare fish behaviour when the host was infected vs. uninfected, whilst also testing whether prior infection status influenced temperature preference.

### *G. turnbulli* temperature tolerance

Parasite-naïve sexually mature experimental fish (>3 months old) were acclimated and maintained at water temperatures of 18, 24 or 32 °C (*n* = 20, 37 and 16, respectively). This entailed increasing or decreasing daily water temperature by 1 °C for 8 or 6 days to reach 32 or 18 °C, respectively. These fish were then maintained for a further 14 days under these conditions before being experimentally infected with two Gt3 worms on their caudal fin (according to the above protocol). Fish were individually housed in 1-L containers and screened daily over a 7-day period to record *Gyrodactylus* infection trajectories.

### Statistical analysis

Analyses were conducted using R statistical software (version 3.1.3, R Development Core Team 2009). Statistical models were refined by deleting non-significant terms from the starting model, based on ANOVA (Crawley 2007). Model robustness was assessed using residual plots (after Pinheiro and Bates 2000).

Using a generalised linear mixed model (GLMM), we investigated whether the mean temperature preference of fish was significantly different before and after infection. Temperature preference was the dependant term in the model and fixed effects included infection status (infected or uninfected), shoal type (female only or mixed sex), infection regime (fish infected at the beginning or second half of a trial) and standard length (millimetres). Fish identity was nested within shoal number and included as a random factor within the model. A negative binomial GLMM (glmmADMB statistical package) was used to investigate the effects of temperature, host standard length (millimetres), the day of infection (i.e. how many days a fish had been infected prior to a particular screen day), on gyrodactylid trajectories over the 7-day infection period. An interaction between temperature and the day of infection was also incorporated into the model, with Fish identity included as a random term.

## Results

### Fish thermal preference

**Fig. 2:**
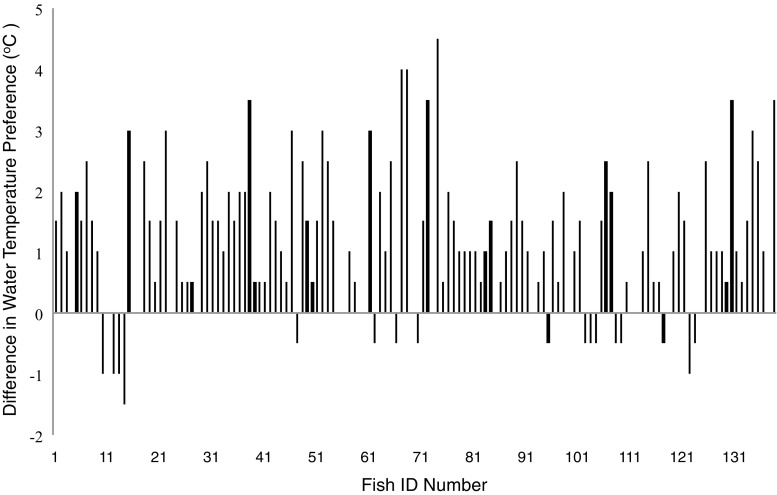
The mean temperature preference of individual fish when infected with *Gyrodactylus turnbulli,* minus their mean temperature preference when uninfected (*n* = 138). *Positive bars* indicate individual fish that moved to warmer waters following infection, *negative bars* indicate individuals that moved to cooler waters when infected

Infection status had a significant effect on the mean temperature preference of fish [GLMM, likelihood ratio test (LRT)_1, 137_ = 819.97, *P* < 0.0001], which was significantly higher when fish were infected (mean = +0.97 °C) than when uninfected (estimate = −1.08, SE = 0.10, *P* < 0.0001; Fig. 2). Shoal type, standard length and infection regime did not influence temperature preference (*P* > 0.05 for all variables).

### *G. turnbulli* temperature tolerance

**Fig. 3:**
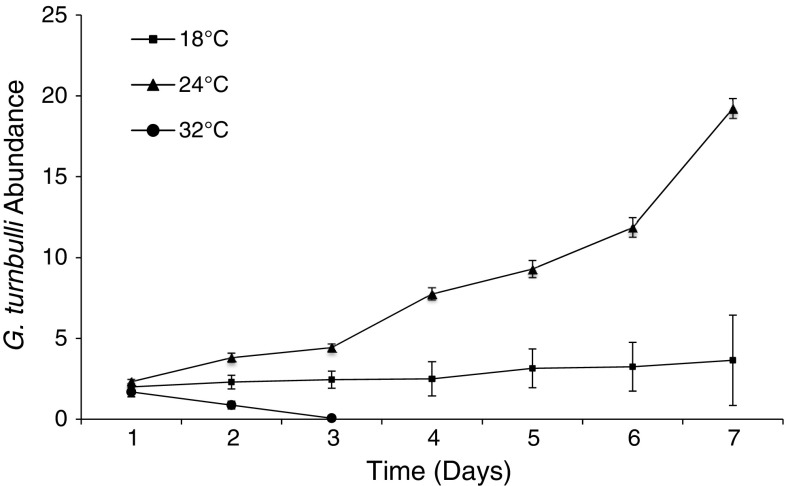
Mean *G. turnbulli* abundance (±SE) on guppies experimentally infected with two parasites on day 0, and maintained at three different temperatures (18, 24 and 32 °C) for a 7-day duration (*n* = 20, 37 and 16, respectively)

Mean parasite abundance significantly increased over the 7-day infection period (GLMM, LRT = 638.70, *df* = 6, *P* < 0.001); however, this was dependant on temperature (LRT = 75.76, *df* = 2, *P* < 0.001). Parasite population increase was higher at 24 °C, compared to 18 °C (estimate = −0.95, SE = 0.22, *P* < 0.0001) and 32 °C (estimate = −3.01, SE = 0.29, *P* < 0.0001) (Fig. 3).

## Discussion

Here, we show for the first time that gyrodactylid-infected fish have a preference for warmer waters, and we speculate that fish exploit the upper thermal tolerances of their parasites to self-medicate against parasite infection. In addition, the guppy immune response may be up-regulated by the increase in temperature, which is consistent with the elevation of lysozyme and immunoglobulin M levels observed in other fish (Bowden et al. 2007). We also confirm the findings of Scott and Nokes (1984) that population growth of *Gyrodactylus turnbulli* is significantly impacted by temperature.

When exposed to a constant temperature, *G. turnbulli* infecting guppies at 32 °C declined to extinction within 3 days. Parasite mean abundance increased on fish maintained at 18 and 24 °C; however, population growth was less pronounced in the 18 °C treatment. Despite *G. turnbulli* population growth being reduced at cooler temperatures, fish residing within such conditions may compromise the metabolic and immunological benefits of warmth. This may explain why, when given a choice of three temperatures (18, 24 and 32 °C), guppies frequented the 32 °C chamber more often compared to when uninfected; this was indicated by a significant increase in mean temperature preference. Three-spined sticklebacks infected with *Schistocephalus solidus* also exhibit a preference for warmer water in comparison to uninfected conspecifics. However, unlike our guppy-gyrodactylid system, this observed thermal shift promotes parasite growth, fecundity and ultimately transmission (Macnab and Barber 2011). The mechanisms involved in this seemingly maladaptive behavioural response are complex and, in the stickleback system, could be affected by both direct and indirect host behavioural manipulation by the parasite (Barber et al. 2004; Scharsack et al. 2007). Although gyrodactylids do cause behavioural changes in their hosts, these are almost certainly by-products of infection rather than parasitic host manipulation (e.g. Kolluru et al. 2009). Therefore, a significant increase in mean temperature preference is likely an adaptive host response. We speculate that this behavioural change directly imposes thermal stress on the parasite to increase mortality, as observed when parasites were maintained at a constant 32 °C, and/or up-regulates the host’s immune system to counteract gyrodactylid infection.

Guppies exhibit innate and acquired resistance to *Gyrodactylus* species; however, little is known about the precise mechanisms involved in guppy immunocompetence (Cable and van Oosterhout 2007b). Although the innate immune response of guppies is probably activated at the onset of *G. turnbulli* infection (Scott 1985; van Oosterhout et al. 2008), parasite population declines are most apparent 7–11 days post-infection at 25 °C. This is presumably associated with the induction of acquired immunity. Our results show that *G. turnbulli* infection did not persist for longer than 3 days on any hosts at 32 °C. The failure of the parasite population at this temperature, particularly in such a short time, indicates that thermal stress, as opposed to the host immune defence, may be the predominant factor compromising parasite survival by impeding physiological function. The parasites used in the current study, however, were not acclimatised to the lower and upper temperature treatments prior to the experiment. Short generation times may facilitate rapid evolution of a wider thermal tolerance within gyrodactylids, although there is no empirical evidence to support this. Due to their small sizes and faster metabolic rates, parasites could acclimate faster than their hosts to thermal shifts, but only if physiological performance is improved at the acclimated temperature (Paull et al. 2015).

Guppies are native to Trinidad and Tobago where they typically reside in warm water ranging between 18–32 °C (Kent and Ojanguren 2015). They have a remarkable capacity for thermal adaptation with populations successfully establishing in environments with very different thermal regimes to their native habitats (Deacon et al. 2011). Water temperatures within freshwater streams can fluctuate by ~10 °C daily (Reeve et al. 2014), and exposure to these temperature heterogeneities often results in marked behavioural changes. Juvenile guppies, for example, increase their average swimming speed and depth when exposed to elevated temperatures (Kent and Ojanguren 2015). Female guppies preferentially associate with larger, more cohesive shoals at high (26 °C) compared to low (22 °C) water temperatures, particularly in the presence of cichlid predators (Weetman et al. 1998, 1999). Although associating with larger shoals may promote gyrodactylid transmission (Richards et al. 2010), by exploiting warmer thermal conditions, fish may self-medicate against parasite infection, particularly monogenean ectoparasites as shown here.

In summary, we use the guppy-*G. turnbulli* model to highlight how elevated temperatures can significantly impact host-parasite interactions within freshwater environments. *G. turnbulli* mean abundance increased at 18 and 28 °C, whilst thermal extremes of 32 °C caused population extinction. Additionally, it is shown how temperature selection by fish is influenced by parasite infection, with infected individuals frequenting warmer water more often than if uninfected. We speculate that this adaptive host behavioural response inhibits physiological functioning of gyrodactylid worms. This information helps us understand how existing natural variation in water temperature, at a local scale, influences disease outbreaks. In the future, we will be able to use such data to model how climate-driven population responses alter disease epidemics in wild and managed fish stocks within both tropical and temperate regions. Temperate species in particular face additional challenges associated with elevated temperature, including oxygen depletion within warmer water that subsequently impedes gill respiratory processes. Whether or not temperate species will tolerate thermal conditions outside their own temperature optima in order to self-medicate against parasite infection remains unknown.
